# Control of Germline Stem Cell Division Frequency – A Novel, Developmentally Regulated Role for Epidermal Growth Factor Signaling

**DOI:** 10.1371/journal.pone.0036460

**Published:** 2012-05-07

**Authors:** Benjamin B. Parrott, Alicia Hudson, Regina Brady, Cordula Schulz

**Affiliations:** 1 Hollings Marine Laboratory, Medical University of South Carolina, Charleston, South Carolina, United States of America; 2 Department of Cellular Biology, University of Georgia, Athens, Georgia, United States of America; 3 Mercer Medical School, Macon, Georgia, United States of America; University of Massachusetts Medical School, United States of America

## Abstract

Exploring adult stem cell dynamics in normal and disease states is crucial to both better understanding their *in vivo* role and better realizing their therapeutic potential. Here we address the division frequency of Germline Stem Cells (GSCs) in testes of *Drosophila* melanogaster. We show that GSC division frequency is under genetic control of the highly conserved Epidermal Growth Factor (EGF) signaling pathway. When EGF signaling was attenuated, we detected a two-fold increase in the percentage of GSCs in mitotic division compared to GSCs in control animals. *Ex vivo* and *in vivo* experiments using a marker for cells in S-phase of the cell cycle showed that the GSCs in EGF mutant testes divide faster than GSCs in control testes. The increased mitotic activity of GSCs in EGF mutants was rescued by restoring EGF signaling in the GSCs, and reproduced in testes from animals with soma-depleted EGF-Receptor (EGFR). Interestingly, EGF attenuation specifically increased the GSC division frequency in adult testes, but not in larval testes. Furthermore, GSCs in testes with tumors resulting from the perturbation of other conserved signaling pathways divided at normal frequencies. We conclude that EGF signaling from the GSCs to the CySCs normally regulates GSC division frequency. The EGF signaling pathway is bifurcated and acts differently in adult compared to larval testes. In addition, regulation of GSC division frequency is a specific role for EGF signaling as it is not affected in all tumor models. These data advance our understanding concerning stem cell dynamics in normal tissues and in a tumor model.

## Introduction

Adult stem cells self-renew and give rise to differentiating daughters that maintain specific tissues throughout the life of an individual. The therapeutic potential of stem cells and the etiological role they may play in cancer biology make studying the behavior of these cells in living animals crucial to our long-term ability to both treat and prevent disease [1], [2]. Over the past two decades, our understanding of how stem cells contribute to tissue homeostasis has increased considerably. Specifically, the physical nature of the microenvironments, the stem cell niches, have been identified for several tissues maintained by stem cells. Furthermore, the developmental pathways regulating the cell fate decisions of stem cells and their daughters to either self-renew or to differentiate have been studied in several model organisms [3]–[5].

Less is known about how the mitotic activity of stem cells is regulated in vivo. This understudied aspect of stem cell biology is crucial because small changes in the frequency of stem cell divisions can dramatically alter the number of terminally differentiated cells. In mammalian tissues, stem cells are generally thought to be long-lived and to cycle slowly [6], [7]. Yet, it is not well understood how the unique cell cycle of stem cells is regulated to ensure that the proper number of differentiated daughter cells are available at any given time. In addition to their role in tissue homeostasis, stem cells have been proposed to play a crucial role in tumor initiation and progression [2]. However, we have yet to gain a full understanding of stem cell behavior in tissues containing tumors. Hence, insights into the stem cell dynamics within tumor bearing tissues may shed light on their oncogenic properties.

**Figure 1:**
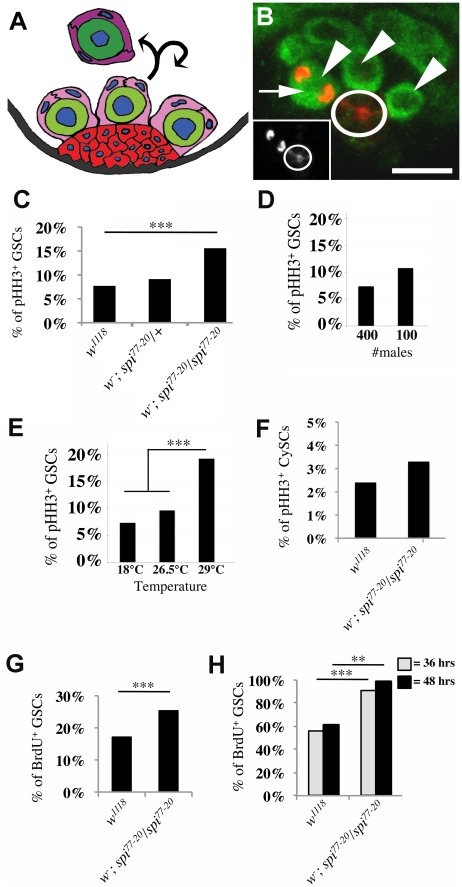
GSCs in *spi^77-20^* testes cycle faster than GSCs in control testes. (A) Cartoon depicting the organization of germ line cells and somatic cells at the tip of wildtype testes. GSCs (light green) are organized around the hub (red). CySCs (light pink) encase GSCs and are also in contact with the hub. The gonialblast (dark green) is displaced away from the hub and encased by two cyst cells (dark pink). (B) The apical tip of a *w^1118^* testis stained with antibodies labeling the cytoplasm of the germline cells (anti-Vasa, green), the membrane of the hub cells (anti-Fasciclin III, red), and mitotic chromatin (anti-pHH3, red). Arrowheads: GSCs, arrow: GSC in mitosis, scale bar: 10 µm. The inset shows the pHH3-positive GSC next to the hub (circle). (C–H) Genotypes as indicated. >500 stem cells were scored for each genotype. (C–E) The percentage of pHH3-positive GSCs (M-phase index). (C) ***p-value<0.0001. (D) Conditions as indicated. p-value = 0.18 (E) Conditions as indicated. ***p-value<0.0001; No significant difference was noted between 18°C and 26.5°C, p-value = 0.22. (F) The percentage of pHH3-positive CySCs. No significant difference was noted. p = 0.28. (G,H) GSC S-phase indices. (G) Ex vivo labeling of testes with BrdU, ***p-value = 0.0004. (H) Flies fed a continuous diet of BrdU for 36 hours or 48 hours, ***p-value<0.0001, **p-value = 0.0074.

Stem cell populations of the *Drosophila* gonad are remarkably similar to those found in vertebrates and studies using this model have revealed fundamental insights into stem cell biology. The *Drosophila* testis is a coiled, tubular structure that contains nine to twelve GSCs at the apical tip which are organized around a group of terminally differentiated somatic cells, termed the hub (Figure 1A, 1B). When a GSC divides, one of the daughter cells maintains contact with the hub and retains stem cell identity, while the other daughter cell is displaced away from the hub and initiates a highly coordinated cascade of differentiation steps [8]–[10]. This well-defined population of GSCs coupled with the genetic tractability of *Drosophila* provides an ideal model to investigate the mechanisms by which stem cell divisions are regulated.

As in mammalian tissues, the differentiation program of a GSC daughter, the gonialblast, begins with transit amplification divisions. A gonialblast undergoes precisely four rounds of transit amplification divisions with incomplete cytokinesis to give rise to exactly 16 interconnected spermatogonia. These cells then differentiate into the spermatocyte stage during which they grow considerably in size and undergo meiotic divisions before they differentiate into spermatids [8], [9].

In the *Drosophila* testis, germline cells are intimately associated with somatic cells that comprise their cellular microenvironment. Each GSC is associated with two Cyst Stem Cells (CySCs) that form cytoplasmic extensions around the GSC and into the hub (Figure 1A) [8]. A tight localization of cell adhesion molecules at the interface of the hub and the GSCs assures the physical contact that is essential for the maintenance of the GSC population [11], [12]. Furthermore, stem cells receive signals from the hub cells. Hub cells secrete the ligand Unpaired (Upd) that induces stem cell identity in the neighboring CySCs via the Janus Kinase-Signal Transducer and Activator of Transcription (Jak-STAT) signaling pathway. CySCs then relay the signal conferring stem cell identity to the encased GSCs via the Transforming Growth Factorβ (TGFβ) signaling pathway [13]–[18]. CySCs also divide asymmetrically to give rise to a renewed CySC and a post-mitotic cyst cell [19]. Two cyst cells form cytoplasmic extensions that completely enclose a newly formed gonialblast and this association is maintained until the final stages of spermatogenesis [8]. Cyst cells comprise the cellular microenvironment for differentiating germline cells and signal to the enclosed germ cells to regulate their differentiation [13], [20]–[22].

Germline tumors in the *Drosophila* testis result from perturbations of several signaling pathways between the germline cells and the somatic cells, including the EGF signaling pathway. Within the germline cells, the EGF ligand, Spitz (Spi), is cleaved into its active, secreted form by the protease Stet [20], [23]. Germline cells in testes from animals harboring mutations in either *spi* or *stet* do not associate with cyst cells and fail to differentiate. Instead, they accumulate as GSCs, gonialblasts, and early stage spermatogonia, resembling germ cell tumors [20], [24]. Other types of germ cell tumors result from the hyperactivation of either the Jak/STAT or the TGFβ signaling pathways. Overexpression of either the Jak/STAT ligand *upd* or the TGFβ ligand *decapentaplegic (dpp)* in germline cells results in their accumulation at early stages. Whereas *upd* overexpression leads to testes filled with single germline cells that resemble GSCs [25], *dpp* overexpression leads to clusters of supernumerary spermatogonia [26], [27]. Although the germline tumor phenotypes arising from EGF attenuation, Jak/STAT hyperactivation, or TGFβ hyperactivation are unique in certain aspects, they can all be classified as overproliferation phenotypes. A unifying theme amongst these overproliferation phenotypes is the failure of germline cells to differentiate past the spermatogonial stage.

Here, we report on the division dynamics of GSCs in response to attenuated EGF signaling. GSCs in EGF mutant testes contained more cells in M-phase and in S-phase of the cell cycle and it took significantly less time for all GSCs within one testes to complete one round of the cell cycle compared to GSCs in control testes. Confirming the role for EGF signaling in regulating the frequency of GSC divisions, germline-specific expression of EGF ligand rescued the hyperproliferation of GSCs in EGF mutant animals. Mutations in *stet* as well as RNAi-mediated knockdown of the EGFR in cyst cells recapitulated the increased mitotic activity of GSCs. These data demonstrate a novel and specific role for EGF signaling: the repression of GSC division frequency.

This novel role for EGF signaling is developmentally independent of its previously reported role in promoting germ cell differentiation [20], [24]. We show that EGF is required to repress the frequency of GSC divisions specifically in adult animals but not during larval stages. This reveals a surprising and substantial bifurcation of EGF function in maintaining the critical balance between GSC division and stem cell daughter differentiation. Finally, we show that GSCs in testes with germline tumors resulting from the hyperactivation of either the TGFβ or the Jak/STAT signaling pathways divided normally. These data show that subsets of hyperplasias are not only characterized by an increase in the number of cycling germ cells, but also by increased mitotic activity of individual stem cells.

## Results and Discussion

### EGF Regulates the Length of the GSC Cell Cycle

To address the in vivo division dynamics of stem cells, we quantified the percentage of GSCs in mitosis (M-phase index). Testes were labeled with a hub marker (anti-Fasciclin III), a germ cell marker (anti-Vasa), and a mitosis marker (anti-phosphorylated Histone-H3 (PHH3), Figure 1B). The M-phase index was then calculated by dividing the number of PHH3-positive, Vasa-positive cells next to the hub by the total number of Vasa-positive cells next to the hub.

We first examined the role of EGF signaling in GSC divisions using testes from animals harboring the temperature sensitive EGF allele, *spi^77-20^*. As previously reported [24], testes from *spi^77-20^* mutant animals grown at a restrictive temperature of 26.5°C were small and filled with early stage germline cells (not shown). We discovered that the M-phase index was approximately two-fold higher for GSCs in testes from *spi^77-20^* mutant animals (*spi^77-20^* testes, 15.6%, n = 854) than the M-phase index for GSCs in *w^1118^* control testes (*w^1118^* testes, 7.7%, n = 1158, Figure 1C).

We noted that the M-phase index for GSCs in control testes underlied fluctuations dependent on several factors. Fluctuations in GSC divisions were previously observed dependent on nutrient availablity and on age of the animals [28]–[31]. Here, we noted that GSCs from males kept at a low population density (100 males/bottle) reproducibly had a slightly higher M-phase index (10%, n = 600) compared to GSCs from males kept at a higher population density (400 males/bottle, 7.5%, n = 600, Figure 1D). Though this difference is not statistically significant, the accumulation of several factors may influence the results from different sets of experiments. Most important, we detected a striking difference in the GSC M-phase index when males were raised and kept at different temperatures (Figure 1E). GSCs from males raised and kept at 29°C had a very high M-phase index (19.3%, n = 467) compared to GSCs from males raised and kept 18°C (7%, n = 521), or 26.5°C (9.5%, n = 578). This difference is extremely statistically relevant, with a p-value below 0.0001. The increase in M-phase index of flies raised and kept at 29°C may not be surprising as the fly metabolism rate may be increased at such a high temperature.

To circumvent fluctuations as much as possible and to be able to compare the GSC division frequencies from different sets of experiments, we only used three to ten day old adult males that were raised and kept at 26.5°C. Prior to dissection, these males were kept at a population density of 100 males/bottle and fed with fresh yeast paste for three days. All data were reproduced in at least three independent experiments. Under these conditions, the range of GSC M-phase indices was highly reproducible. The GSC M-phase index from control testes always ranged between 6% and 10% (n>300 GSCs/experiment). The M-phase index of *spi^77-20^* testes, in contrast, always ranged significantly higher (>15%, n>300 GSCs/experiment) than the M-phase index observed for GSCs in control testes.

In contrast to the GSCs, CySCs did not have an increased M-phase index in *spi^77-20^* testes. We hypothesized that GSC and CySC divisions may be coordinated to ensure that two cyst cells are produced for each gonialblast. CySCs and their daughters associated with early stage spermatogonia express the transcription factor Traffic jam (Tj) in their nuclei [32]. Tj-positive, pHH3-positive CySCs are located within one cell diameter away from the hub. Therefore, by measuring the percentage of Tj-positive, pHH3-positive cells we were able to calculate the M-phase index of CySCs. Interestingly, the M-phase index of CySCs in *spi^77-20^* testes (3.3%, n = 602) was similar to that of CySCs in *w^1118^* testes (2.4%, n = 1120, Figure 1F), suggesting that different pathways regulate the division frequency of the two stem cell populations.

To determine how loss of EGF affects other phases of the cell cycle of GSCs, we quantified the percentage of GSCs in S-phase (S-phase index) using ex vivo labeling with the thymidine analog, BrdU. We found that the S-phase index of GSCs from *spi^77-20^* testes (25.3%, n = 843) was significantly higher than the S-phase index calculated for GSCs from *w^1118^* testes (17.1%, n = 521, Figure 1G). Together, our data suggest that GSCs in *spi^77-20^* testes either underwent a shorter cell cycle, or that mitosis and synthesis occupied a larger proportion of the cell cycle in GSCs from *spi^77-20^* testes compared to controls. To address this question, we measured the total length of the cell cycle by in vivo labeling with BrdU. We reasoned that if the GSCs in *spi^77-20^* testes indeed divide faster than the GSCs in *w^1118^* testes, then it should take a shorter time until all the GSCs underwent division and were positive for BrdU. After 36 hours, the S-phase index of GSCs in *spi^77-20^* testes (91%, n = 80 testes) was already dramatically higher than the S-phase index of GSCs in *w^1118^* testes (56%, n = 118 testes, Figure 1H). After 48 hours, almost all of the GSCs in *spi^77-20^* testes had detectable BrdU incorporation (99%, n = 141 testes), whereas only 61% (n = 144 testes) of GSCs from the *w^1118^* testes had detectable BrdU incorporation (Figure 1H). This strongly suggests that mutations in EGF shorten the cell cycle, thereby increasing GSC division frequency.

Our S-phase indices are consistently lower than those reported in a previous study [31]. Here, we present the S-phase indices from a large number of GSCs and the differences between genotypes are highly statistically relevant (see p-values in Figure legends). Using BrdU ex vivo labeling, we calculated each of the S-phase indices from GSCs in 300 testes (compared to the S-phase index from GSCs in 20 testes presented in the previous study). Similarly, using BrdU in vivo labeling, we calculated the S-phase indices from GSCs in 80 to 144 testes for the different points in time (compared to 10 testes observed in the previous study). Given the above described fluctuations in M-phase indices and the different scale of our study, it is not surprising that we report different S-phase indices. However, for both studies the differences between genotypes appear striking and biologically relevant.

Small changes in the frequency of stem cell divisions can have a dramatic effect on cell number and tumor growth. Thus, it is not surprising that mechanisms have evolved to regulate this aspect of stem cell biology. EGF-dependent regulation of GSC division frequency may be important for reducing the frequency of unnecessary cell divisions that increase the chance of mutations being introduced into the germline. Alternatively, it may simply be required for increasing the duration of fitness by mobilizing energy away from sperm production.

### EGF Signals to the Soma to Repress GSC Divisions

**Figure 2:**
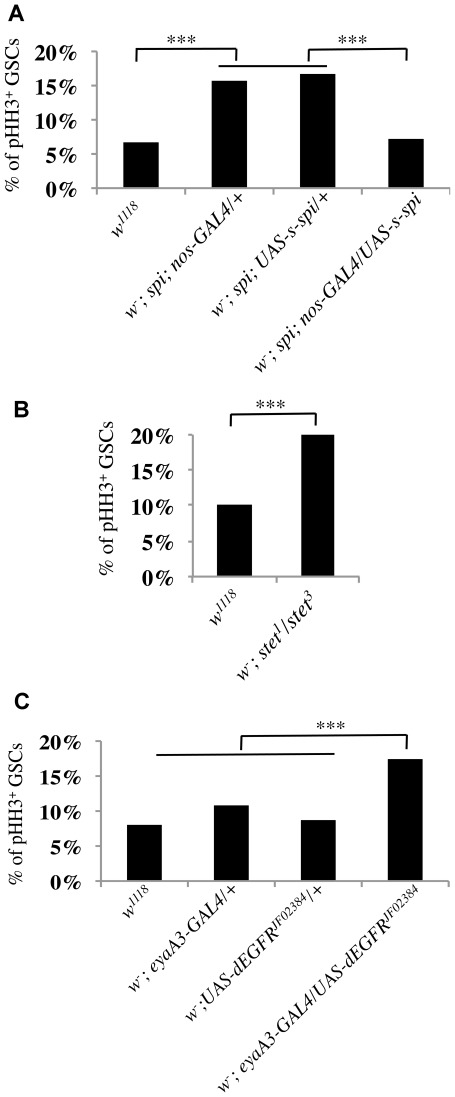
EGF signaling from the germline to the soma decreases the frequency of GSC divisions. (A–C) The percentage of pHH3-positive GSCs for each indicated genotype. (A) The expression of *s-spi* in germ cells rescues the hyper-proliferation of GSCs in *spi^77-20^* testes. (B) GSCs in *stet^1^/stet^3^* testes showed an increased M-phase index compared to *w^1118^* testes. (C) RNAi-mediated knock-down of EGFR in the soma causes a higher GSC M-phase index, ***p-value≤0.0001.

Expression of a secreted form of EGF, s-Spi, specifically in the germline cells restores spermatogenesis in *spi^77-20^* testes [24]. We found that the M-phase indices of GSCs in *spi^77-20^* testes from animals carrying either the *nanos-*Gal4- (15.7%, n = 1060) or the UAS-*s-spi*-constructs (16.7%, n = 1157) alone were approximately two-fold higher than those calculated for GSCs from *w^1118^* testes (6.7%, n = 669) (Figure 2A). In contrast, GSCs in *spi^77-20^* testes from animals carrying both the *nanos-*Gal4*-* and the UAS-*s-spi-*construct had a M-phase index (7.2%, n = 833) similar to that observed from GSCs in *w^1118^* testes (Figure 2A). These data confirm that the increased division frequency of GSCs was due to the reduction of EGF in *spi^77-20^* testes.

Mutations in the germline specific protease Stet [20], [33] also resulted in GSCs with an increased M-phase index. Since animals carrying strong *stet* alleles do not have intact testis sheaths [20], we quantified the GSC M-phase index in testes from a hypomorphic *stet*-allele, *stet^3^*, over an amorphic allele, *stet^1^*. We found that the M-phase index of GSCs in testes from *stet^1^/stet^3^* males was increased (19.9%, n = 607) relative to GSCs in *w^1118^* testes (10.1%, n = 424) (Figure 2B). Similarly, expression of a transgenic RNAi construct targeted specifically against dEGFR (UAS-*dEGFR^JF02384^*) in CySCs and cyst cells increased GSC division frequency. We used the UAS/Gal4-system [34], [35] to reduce the EGFR specifically from the germline, or from the soma. Gal4 is most active at a temperature of 29°C [35]. However, to keep the conditions among different sets of experiments constant, the flies were raised and kept at 26.5°C. Even at 26.5°C, testes with expression of UAS-*dEGFR^JF02384^* using the soma-specific *eyaA3-*Gal4 transactivator displayed all defects characteristic of the loss of EGF signaling (data not shown), including a higher M-phase index (17.4%, n = 1222) compared to the GSC M-phase indices calculated from control animals carrying either the *eyaA3-*Gal4- (10.8%, n = 944) or the UAS-*dEGFR^JF02384^*-construct (8.7%, n = 620) alone (Figure 2C). Expression of UAS-*dEGFR^JF02384^* in the germline or the somatic hub cells did not result in increased M-phase indices (data not shown). These data strongly suggest that GSC-secreted EGF is received via the EGFR on CySCs, and that this signaling event in turn represses the frequency of GSC divisions.

### The Role of EGF in Repressing GSC Division Frequency is Developmentally Regulated

**Figure 3:**
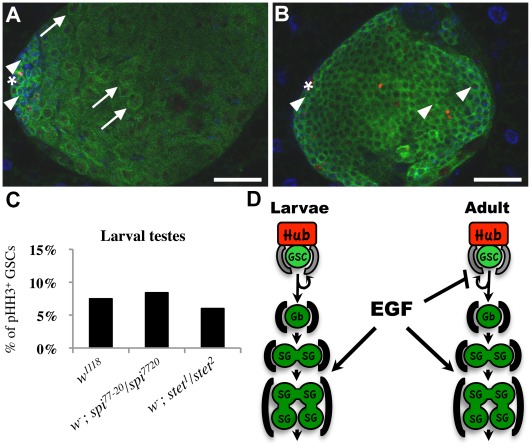
The EGF repression of GSC divisions is developmentally regulated. (A, B) Testes from (A) *w^1118^* or (B) *w^-^; spi^77-20^/spi^77-20^* 3^rd^ instar larvae stained with anti-Vasa (green) and DAPI (blue). Arrows: spermatocytes, arrowheads: early stage germline cells, scale bars: 50 µm. (C) M-phase index for the GSCs of each genotype. No significant difference was detected, all p**-**values>0.30. (D) A model depicting the requirement for EGF signaling. EGF is required in both larvae and adults for promoting germline differentiation. In contrast, EGF is not required in larvae for the repression of GSC division frequency, demonstrating a developmental uncoupling of EGF-function.

To gain insights into how stem cell behavior is governed during development, we investigated the division frequency of GSCs in 3^rd^ instar larvae and adults. The testes of *Drosophila* third instar larvae are round discs that have yet to undergo the morphogenetic events that result in a coiled tube connected to the reproductive tract and genitalia. Although *Drosophila* males do not reach sexual maturity until after eclosion, spermatogenesis begins during the 1^st^ instar of larval development. By the end of the 3^rd^ larval instar, testes contain germline cells in most stages of spermatogenesis (Figure 3A), occasionally including elongated spermatids [9]. Similar to the phenotype of adult *spi^77-20^* testes, larval *spi^77-20^* testes were filled with early stage germline cells and lacked more mature germline cells (Figure 3B). We reasoned that if EGF is required for germline cells to adopt late stage cell fates in larval testes then GSCs in *spi^77-20^*-testes might also hyperproliferate during this stage. However, the M-phase index of GSCs in larval *spi^77-20^* testes (8.4%, n = 733) was similar to that of larval *w^1118^* testes (7.5%, n = 702, Figure 3C).

To further rule out the possibility that low levels of persisting Spi activity were sufficient to repress the frequency of GSC divisions in larval *spi^77-20^* testes, we measured the M-phase index of GSCs in larvae mutant for *stet.* Since the allelic combination *stet^1^/stet^3^* gave rise to an increased M-phase index in adult testes, we reasoned that we should observe an effect on GSC division frequency with the stronger *stet^1^* and *stet^2^* allelic combination. Just as in larval *spi^77-20^* testes, the M-phase index of GSCs in larval testes from *stet^1^/stet^2^* mutant animals (5.8%, n = 495) was similar to the mitotic index of GSCs in larval *w^1118^* testes (Figure 3C). We conclude that EGF signaling has two differentially regulated functions in *Drosophila* spermatogenesis: to promote stem cell daughter differentiation in both larvae and adults, and to repress the frequency of GSC divisions in adults, but not in larvae.

Based on our findings, we propose a model that demonstrates the bifurcation of the EGF signaling pathway (Figure 3D). EGF acts in the CySCs in one pathway to regulate GSC division frequency and in a different pathway in cyst cells to promote germ cell enclosure and differentiation. This developmental bifurcation of EGF function during *Drosophila* spermatogenesis reveals a fundamental uncoupling between the control of stem cell proliferation and the control of stem cell daughter differentiation. The stage-specific requirement for EGF may reflect the different functions of GSCs in immature versus mature tissues. The initial function of GSCs may be to quickly populate larval testes with germline cells, while GSCs in adult testes need to replenish differentiated cells dependent on demand.

Our study is the first report of a stage-specific impact of a signaling pathway on the activity of GSCs and suggests that this developmental switch in GSC activity between larval and adult stages requires the activities of stage-specific pathways. On a molecular level, additional pathways may be active during larval stages that counteract the increased division frequency observed in adult GSCs upon loss of EGF. In larval testes, nutrient availability and cell growth may be the primary factors governing the frequency of GSC divisions. Conversely, soon after eclosion, *Drosophila* males reach sexual maturity and spermatogenesis may rely on EGF-mediated signaling to regulate GSC divisions.

### The Regulation of GSC Division Frequency is Specific to EGF Signaling

**Figure 4:**
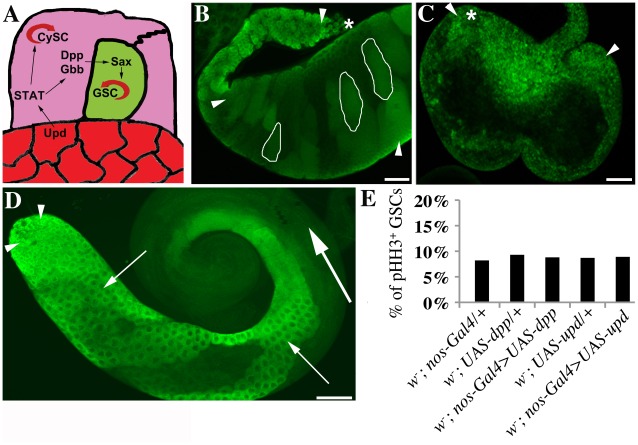
Regulation of GSC division frequency is specific to EGF signaling. (A) Graphic demonstrating how Jak/STAT and TGFβ signaling regulate stem cell fate. Upd: unpaired, Dpp: decapentaplegic, Gbb: glass bottom boat, Sax: Saxophone. (B–D) Testes stained with anti-Vasa. (B,C) Testes with germ cell-intrinsic overexpression of (B) *dpp* contain excessive clusters (white circles) of small early germline cells and (C) *upd* iare filled with small germline cells. (D) Image of a control *w^1118^* testis. Asterisks: apical tips of testes, arrowheads: early stage germline cells, short arrows: spermatocytes, long arrow: elongated spermatids, scale bars: 50 µm. (E) Graph showing the percentage of pHH3-positive GSCs (M-phase index), genotypes as indicated. >500 stem cells were scored for each genotype. No significant differences were observed, all p-values>0.20.

We next addressed whether germline tumors resulting from perturbations tof Jak/STAT or TGFβ signaling also displayed an increased M-phase index. Both signaling pathways are required for GSC fate (Figure 4A). As expected, the overexpression of *dpp* (Figure 4B) or *upd* (Figure 4C) in germline cells resulted in testes with germline tumors. Testes were filled with small germline cells that are normally found only at the tip of wildtype testes (Figure 4D, arrowheads). Although the expected phenotypes were present, the M-phase indices of GSCs from animals overexpressing *dpp* (*nanos*-Gal4>UAS-*dpp*) or *upd* (*nanos*-Gal4>UAS-*upd*) within their germline cells were similar to the M-phase indices of GSCs in testes from control animals harboring either only the *nanos*-Gal4-, the UAS-*dpp-*, or the UAS-*upd-*construct (Figure 4E). We conclude that the mitotic hyperactivity of GSCs is not a hallmark of all hyperplastic phenotypes, but is specifically associated with a reduction in EGF signaling. Furthermore, the observation that GSCs in testes with overexpression of *dpp* or *upd* displayed normal GSC division frequencies argues against a hypothesis in which more mature germline cells, which were lacking in these testes, send retrograde signals to the GSCs for regulating their division frequencies.

Our findings demonstrate two distinct modes of hyperplasic growth in tumors. On one hand, cells continue to proliferate instead of undergoing differentiation. On the other hand, single cells divide more frequently. Although it appears that the failure of cells to differentiate is a general characteristic of germ cell tumors, the mitotic hyperactivity of individual stem cells is specific to a subset of tumors. Among the genetic backgrounds we tested, only the attenuation of EGF signaling led to an increase in the frequency of GSC division. This increase in the frequency of GSC division may lead not only to the production of more proliferating cells, thereby increasing tumor size, but also may lead to an increased risk of stem cells accumulating transforming mutations. Tumors that contain hyperproliferating stem cells in addition to a blockade in differentiation may be more aggressive than tumors consisting primarily of partially differentiated cells. Our comparison of these tumor types strengthens the underlying rationale for alternative treatment options for different types of tumors.

## Materials and Methods

### *Drosophila* Genetics

All fly stocks were raised and maintained on standard cornmeal molasses agar medium at 26.5°C. Mutations and transgenic elements are described in [36] or in the appropriate references provided below. Fly stocks used in this study include: *w^1118^*, UAS-*upd,* UAS-*dpp*, *spi^77-20^* [24], *stet^1^*, *stet^2^*, and *stet^3^* [20], the germ cell driver *nanos-gal4-VP16* [37], the cyst cell driver *eyaA3-*Gal4 [17], UAS*s-spi* [38], and UAS-*dEGFR^JF02384^* (TRiP at Harvard Medical School). All UAS-Gal4 expression studies [34], [35] were performed at 26.5°C.

### Immunohistochemistry, BrdU Labeling, and Fluorescence Microscopy

Testes were dissected and placed in Testis Isolation Buffer (10 mM Tris-HCl, pH 6.8, 180 mM KCl) on ice. Testes were subsequently fixed in 4% formaldehyde in PBT for 30 minutes. Primary antibody incubation took place overnight at 4°C and secondary antibody incubation took place for 2 hours at room temperature. Testes were mounted onto slides using Vectashield mounting medium with DAPI. Tissues were observed using a Zeiss Axiophot microscope. Images were taken with a CCD camera using an Apotome and Axiovision Rel Software. Antibodies and dilutions used were as follows: goat anti-Vasa (1∶100, Santa Cruz Biotechnology Inc.), rabbit anti-phosphorylated Histone-H3 Ser10 (1∶500, Millipore), mouse anti-BrdU (1∶20, Upstate), and anti-FasiciclinIII 7G10 (1∶10, obtained from the Developmental Studies Hybridoma Bank, developed under the auspices of the NICHD, and maintained by The University of Iowa, Department of Biological Sciences, Iowa City, IA 52242: developed by C. Goodman). Alexa-488-, Cy3-, and Cy5-conjugated secondary antibodies were used at 1∶1000 (Invitrogen).

BrdU ex vivo labeling of GSCs was performed as described by [31] with minor differences. Testes were dissected into 10 µM BrdU in Testes Isolation Buffer on ice. Testes were then shifted to 26.5°C on a rotating platform for 30 minutes before being fixed in 4% formaldehyde in PBT for 30 minutes.

For BrdU in vivo labeling, animals were fed 10 µM BrdU in yeast paste and the plates were replaced every 12 hours. Flies were kept at 26.5°C and dissected after 36 or 48 hours.

### Cell Cycle Analysis

All experiments were performed on flies raised and kept at 26.5°C. Adult flies were less than ten days old kept in bottles at a density of 100 males per bottle, and fed with fresh yeast paste for three days prior to dissection. The S-phase and M-phase indices were calculated by dividing either the number of BrdU-positive (S-phase) or pHH3-positive (M-phase) GSCs by the total number of GSCs scored. Optical sections were taken, using an apotome in conjunction with Axiovision Software, of the focal plane in which the middle of the hub was detected. We counted an average of three GSCs in the focal plane scored for each testis. All indices represent the cumulative total of three independent experiments. All p-values were calculated using a two-tailed Fisher’s exact test.
